# The histological analysis of the coronary medial thickness: Implications for percutaneous coronary intervention

**DOI:** 10.1371/journal.pone.0283840

**Published:** 2023-03-31

**Authors:** Takao Konishi, Saikat Kumar B. Ghosh, Yu Sato, Rika Kawakami, Kenji Kawai, Aimee E. Vozenilek, Weili Xu, Arielle Bellissard, Robert Giasolli, Diljon Chahal, Renu Virmani, Aloke V. Finn

**Affiliations:** 1 Department of Cardiovascular Pathology, CVPath Institute, Gaithersburg, MD, United States of America; 2 Cagent Vascular, Wayne, PA, United States of America; 3 School of Medicine, University of Maryland, Baltimore, MD, United States of America; BSMMU: Bangabandhu Sheikh Mujib Medical University, BANGLADESH

## Abstract

**Background:**

A deeper understanding of coronary medial thickness is important for coronary intervention because media thickness can limit the safety and effectiveness of interventional techniques. However, there is a paucity of detailed data on human coronary medial thickness so far.

**Materials and methods:**

We investigated the thickness of the media by histologic analysis. A total of 230 sections from 10 individuals from the CVPath autopsy registry who died from non-coronary deaths were evaluated. We performed pathological analysis on 13 segments of the following primary vessels from coronary arteries: the left main trunk, proximal left anterior descending artery (LAD), mid LAD, distal LAD, proximal left circumflex artery (LCX), mid LCX, distal LCX, proximal right coronary artery (RCA), mid RCA, and the distal RCA. The following side branches were also evaluated: diagonal, obtuse margin, and posterior descending artery branches.

**Results:**

The average age of the studied individuals was 60.4±12.3 years. The median medial thickness for all sections was 0.202 (0.149–0.263) mm. The median medial thickness of the main branches was significantly higher compared to that of the side branches (p<0.001). Although the medial thicknesses of the main branch of LAD and LCX were significantly decreased from proximal to distal segments (p = 0.010, p = 0.006, respectively), the medial thickness of the main branch of RCA was not significantly decreased from proximal to distal (p = 0.170). The thickness of the media was positively correlated with vessel diameter, while it was negatively correlated with luminal narrowing (p<0.001 and p<0.001, respectively).

**Conclusions:**

The human coronary arteries demonstrate variation in medial thickness which tends to vary depending upon an epicardial coronary artery itself, as well as its segments and branches. Understanding these variations in medial thickness can be useful for both the interventionalists and interventional product development teams.

## Introduction

The coronary arteries are composed of three layers: the intima, media, and adventitia. Many previous studies have performed detailed investigations of the ‘intima’ as it is where the first stages of atherosclerosis begin [1, 2]. The media extends from the internal elastic lamina (IEL) to the adventitia and is composed of smooth muscle cells and extracellular matrix. The media is the most important structure of the artery and ensures structural stability. Many imaging studies have performed measurements of medial thickness for peripheral arteries, such as carotid and femoral arteries, using various imaging modalities [3–6]. However, detailed measurements of the thickness of the coronary artery ‘media’ have not been performed, because it is often difficult to visualize the whole media by current coronary imaging devices, such as intravascular ultrasound and optical coherence tomography, due to the shadowing caused by calcification or lipid-rich plaque.

Advancements in percutaneous coronary interventions (PCI) have permitted excellent procedural success rates for complex lesions, including highly calcified lesions and chronic total occlusion (CTO) lesions. These technologies include atherectomy, rotational (Rotablator^®^) or orbital (Diamondback 360^®^), intravascular lithotripsy for severe calcification, excimer laser for in-stent restenosis or thrombotic lesions, antegrade dissection, and the reentry device (Stingray^®^) for CTO lesions. However, a potentially fatal complication from using such devices is coronary perforation, which can lead to critical conditions, including cardiac tamponade [7, 8]. As coronary perforation or rupture usually occurs when a PCI device such as a wire, a balloon catheter, a stent, an ablation catheter, a lithotripsy catheter, or an excimer laser catheter travels outside the media, it is important to recognize which artery, branch, or segment has a thinner coronary media and poses a higher risk of coronary perforation. Moreover, pathological studies suggest that medial damage or disruption is one of the predictors of restenosis after stent implantation [9, 10]. Therefore, there has been a growing need for detailed knowledge of the medial thickness to help improve the safety of complex PCI procedures.

Accordingly, the aim of this study was to investigate the thickness of the media by histologic analysis.

## Materials and methods

### Study population

We randomly selected 10 patients (5 males and 5 females) who died of non-coronary artery disease with a luminal narrowing of less than 70% from the Office of the Chief Medical Examiner (OCME) of the State of Maryland. Clinical records were reviewed for each case. The protocol for the study was approved by the institutional review board of CVPath Institute (RP128). A waiver of consent was granted by the institutional review board because this used only autopsy material.

### Histopathological analysis

Each coronary artery was radiographed and decalcified in 10% EDTA using microwave-assisted decalcification. Following decalcification, the arteries were sectioned at 5 mm intervals along the length of the vessel. Paraffin-embedded coronary arteries were stained using Hematoxylin and eosin and Movat stains. Depending on the vessel length, a series of sections from 1 to a maximum of 3 sections (starting proximally) were included in the analysis. We classified the sections into 13 segments of coronary arteries as follows: main branches: left main trunk (LMT), proximal left anterior descending artery (LAD), mid LAD, distal LAD, proximal left circumflex artery (LCX), mid LCX, distal LCX, proximal right coronary artery (RCA), mid RCA, distal RCA, and the side branches, including the diagonal, obtuse margin (OM), and posterior descending artery (PDA) branches. The thickness of the media was measured at locations with relatively normal media i.e., where there was the least intimal thickening and no atherosclerosis. The measurements were performed at four locations for each section. The measurements of the coronary medial thickness were performed using computerized planimetry (Zen 2.3, Carl Zeiss, Oberkochen, Germany). The tapered ratio was calculated as a value of the difference between the proximal and distal segment diameter (millimeter), divided by the proximal segment diameter (millimeter) and the distance (centimeter) from the proximal segment to the distal segment. Therefore, the tapered ratio was defined as a percent of decreased vessel diameter per longitudinal length.

### Statistical analysis

Normality of distribution was examined by the Shapiro–Wilk test. The results for continuous variables with normal distribution were expressed as mean ± SD. Variables without normal distribution were indicated as median and 25% to 75% interquartiles. Categorical data sets were analyzed by the Chi-square test or by Fisher’s exact tests. Comparisons of continuous variables with normal distribution were performed by the student *t-*test, while the Kruskal-Wallis test was used to analyze the significant differences for variables with non-parametric distribution. A p-value of <0.05 was considered statistically significant. All statistical analysis was performed using JMP software (version 15.0, SAS, Cary, NC).

## Results

### Patient and lesion characteristics

The patient characteristics are shown in S1 Table. The average age at the time of death was 60.4 ± 12.3 years. The lesion characteristics are shown in Table 1. A total of 230 sections were analyzed. 190 (83%) sections from the main branches and 40 (17%) sections from the side branches were analyzed. The median luminal narrowing was 40.2% [interquartile range (IQR) 24.4%-54.2%]. The morphological plaque analysis showed that the frequencies of adaptive intimal thickening, pathological intimal thickening, fibroatheroma and fibrocalcific plaque were 47%, 27%, 2% and 23%, respectively.

**Table 1:** Patient and lesion characteristics.

Section, n	n = 230
Branch	
Main/side	190 (83)/40 (17)
Vessel	
LMT/LAD/LCX/RCA	19 (8)/67 (29)/68 (30)/76 (33)
Segment of each vessel	
Proximal/mid/distal	78 (46) /48 (28)/ 45 (26)
Lesion characteristics	
Luminal narrowing (%)	40.2 (24.4–54.2)
Vessel diameter, mm	2.90±0.96
Adaptive intimal thickening	109 (47)
Pathological intimal thickening	62 (27)
Fibroatheroma	5 (2)
Fibrocalcific plaque	54 (23)

Continuous variables are presented as mean ± standard deviation if normally distributed and median (interquartile range) if not normally distributed. LMT, left main trunk; LAD, left anterior descending artery; LCX, left circumflex artery; RCA, right coronary artery (RCA); OM, obtuse margin (OM) branch; PDA, posterior descending artery branch.

### The measurements of vessel diameter and medial thickness

**Fig 1:**
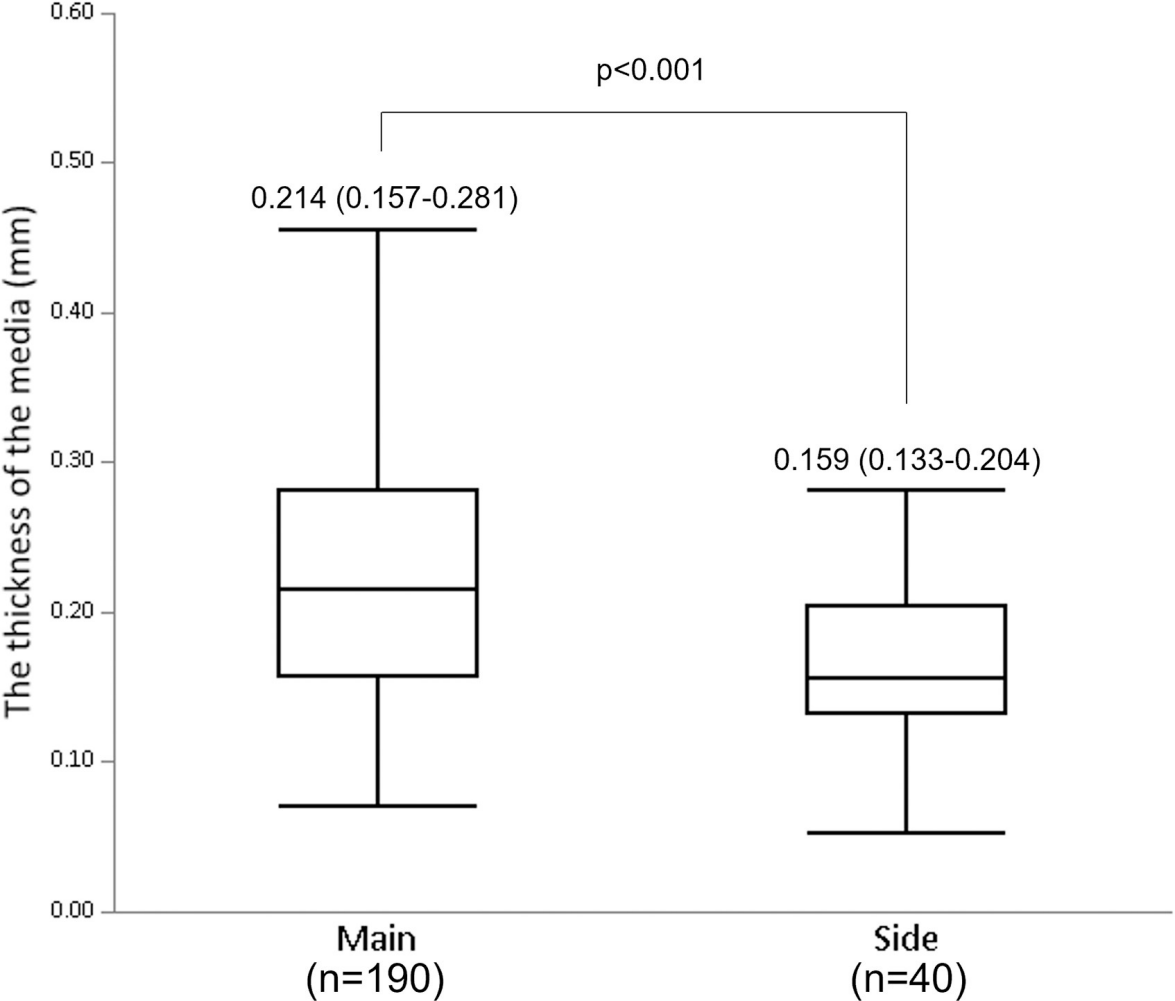
The comparison of the medial thicknesses between main and side branches. The median thickness of the media in the main branches was significantly higher than in the side branches (0.214 mm [0.157–0.281mm] vs 0.159 mm [0.133–0.204 mm], respectively, p<0.001).

**Fig 2:**
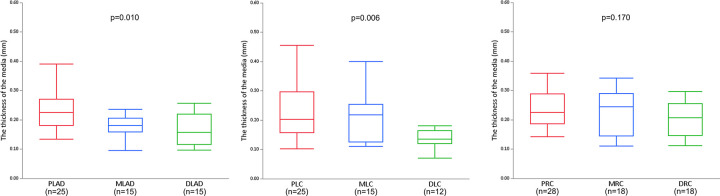
The comparison of the medial thicknesses between proximal, mid, and distal segments of LAD, LCX, and RCA. **A.** The medial thickness of the main branch of LAD became lower from proximal to distal segments (p = 0.010). **B.** The medial thickness of the main branch of LCX became lower from proximal to distal segments (p = 0.006). **C.** The medial thickness of the main branch of RCA did not significantly become lower from the proximal to distal segments (p = 0.170). LAD, left anterior descending artery; PLAD, proximal LAD; MLAD, mid LAD; DLAD, distal LAD. LCX, left circumflex artery; PLC, proximal LCX; MLC, mid LCX; DLC, distal LCX. RCA, right coronary artery; PRC, proximal RCA; MRC, mid RCA; DRC, distal RCA.

The measurements of the vessel diameter and the medial thickness for each coronary artery segment are shown in Table 2. The average vessel diameter and median medial thickness for all sections were 2.90±0.96 mm and 0.202 (0.149–0.263) mm, respectively. The median medial thickness of the main branches was significantly higher compared to that of the side branches (0.214 mm [0.157–0.281 mm], 0.159 mm [0.133–0.204 mm], respectively, p<0.001) (Fig 1). The medial thicknesses of the main branch of LAD and LCX were getting significantly lower toward the distal segments (proximal LAD: 0.235±0.071 mm vs. mid LAD: 0.184±0.057 mm vs. distal LAD: 0.157 mm [0.117–0.215 mm], p = 0.010; and proximal LCX: 0.227±0.092 mm vs. mid LCX: 0.216±0.088 mm vs. distal LCX: 0.134 mm [0.125–0.155 mm], p = 0.006, respectively) (Fig 2A and Fig 2B). However, the medial thickness of the distal RCA was not significantly lower compared to that of the mid and proximal RCA (proximal RCA: 0.225 mm [0.189–0.284 mm] vs. mid RCA: 0.223±0.072 mm vs distal RCA: 0.200±0.059 mm, p = 0.170) (Fig 2C). The tapered ratio was significantly lower in RCA (3.5%±2.1%/cm), compared to LAD (8.2%±2.7%/cm) or LCX (9.7%±1.3%/cm) (p<0.001 and p<0.001, respectively) (S1 Fig). To confirm whether there were any differences in the severity of atherosclerosis between the studied vessels, we compared the luminal narrowing and plaque type. The luminal narrowing and each plaque type for the main and side branches were not statistically different (S2 Table). Similarly, luminal narrowing and each plaque type for LAD, LCX and RCA were not significantly different between the proximal, mid, and distal segments (S3–S5 Tables).

**Table 2:** Measurements of the vessel diameter and the medial thickness.

Segment (sections)	Vessel diameter (mm)	Medial Thickness (mm)
Total (n = 230)	2.90±0.96 mm	0.202 (0.149–0.263)
Main branch		
LMT (n = 19)	3.94 (3.54–4.33)	0.362±0.099
Proximal LAD (n = 25)	3.70±0.79	0.235±0.071
Mid LAD (n = 15)	2.70 (2.56–3.56)	0.184±0.057
Distal LAD (n = 15)	1.88±0.72	0.157 (0.117–0.215)
Proximal LCX (n = 25)	3.22±0.86	0.227±0.092
Mid LCX (n = 15)	3.06±0.94	0.216±0.088
Distal LCX (n = 12)	1.99 (1.76–2.11)	0.134 (0.125–0.155)
Proximal RCA (n = 28)	3.40±0.55	0.225 (0.189–0.284)
Mid RCA (n = 18)	2.99±0.38	0.223±0.072
Distal RCA (n = 18)	2.66±0.31	0.200±0.059
Side branch		
Diagonal (n = 12)	2.53 (2.18–2.62)	0.172±0.055
OM (n = 16)	2.27±0.87	0.172±0.068
PDA (n = 12)	1.71±0.34	0.167±0.038
Total (all sections) (n = 230)	2.90±0.96	0.202 (0.149–0.263)

Continuous variables are presented as mean ± standard deviation if normally distributed and median (interquartile range) if not normally distributed. LMT, left main trunk; LAD, left anterior descending artery; LCX, left circumflex artery; RCA, right coronary artery (RCA); OM, obtuse margin (OM) branch; PDA, posterior descending artery branch.

### The relationships between the medial thickness, the vessel diameter, and the luminal narrowing

**Fig 3:**
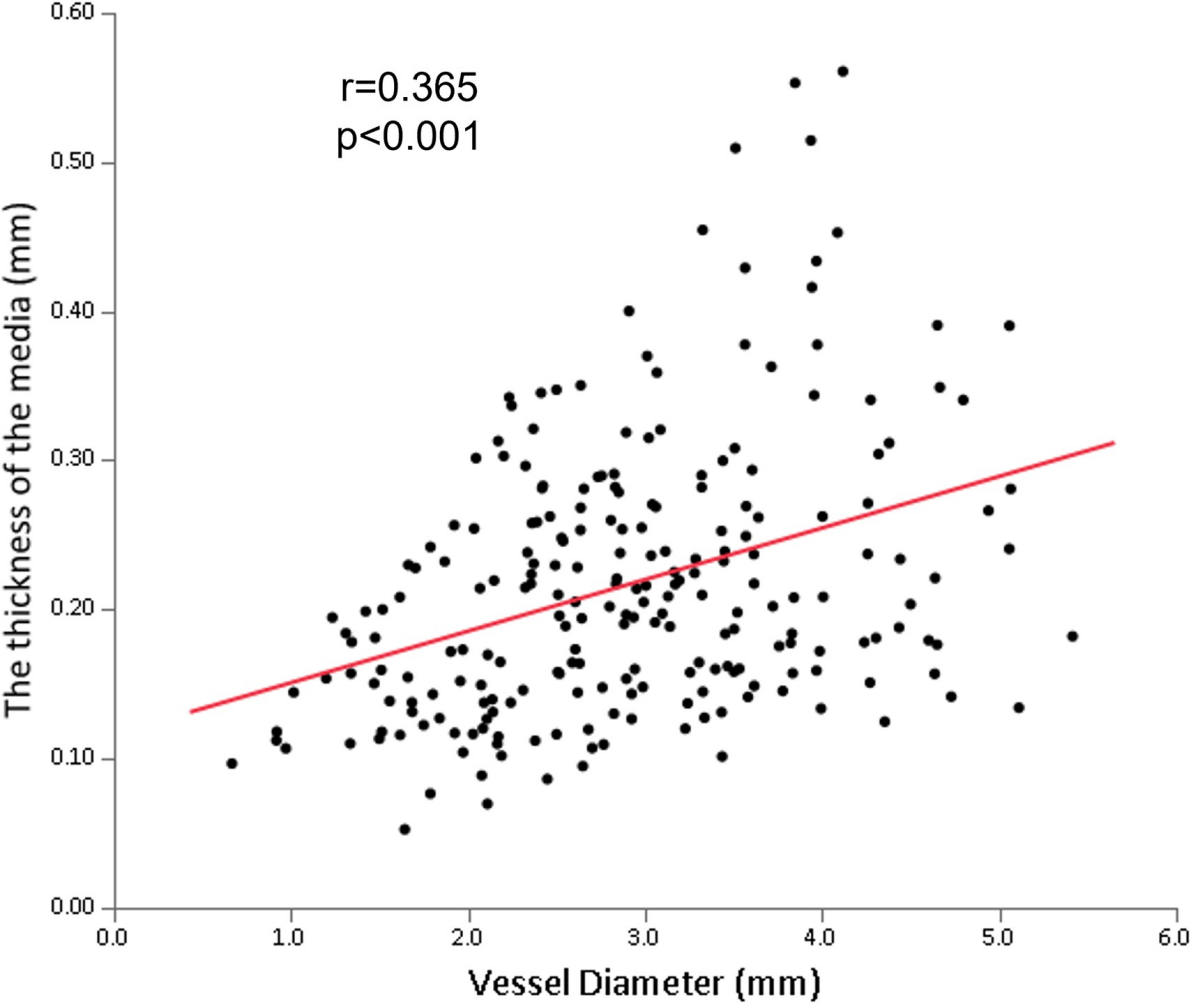
The relationship between the medial thicknesses and the vessel diameter. The thickness of the media directly correlated with the vessel diameter (r = 0.365, p<0.001).

**Fig 4:**
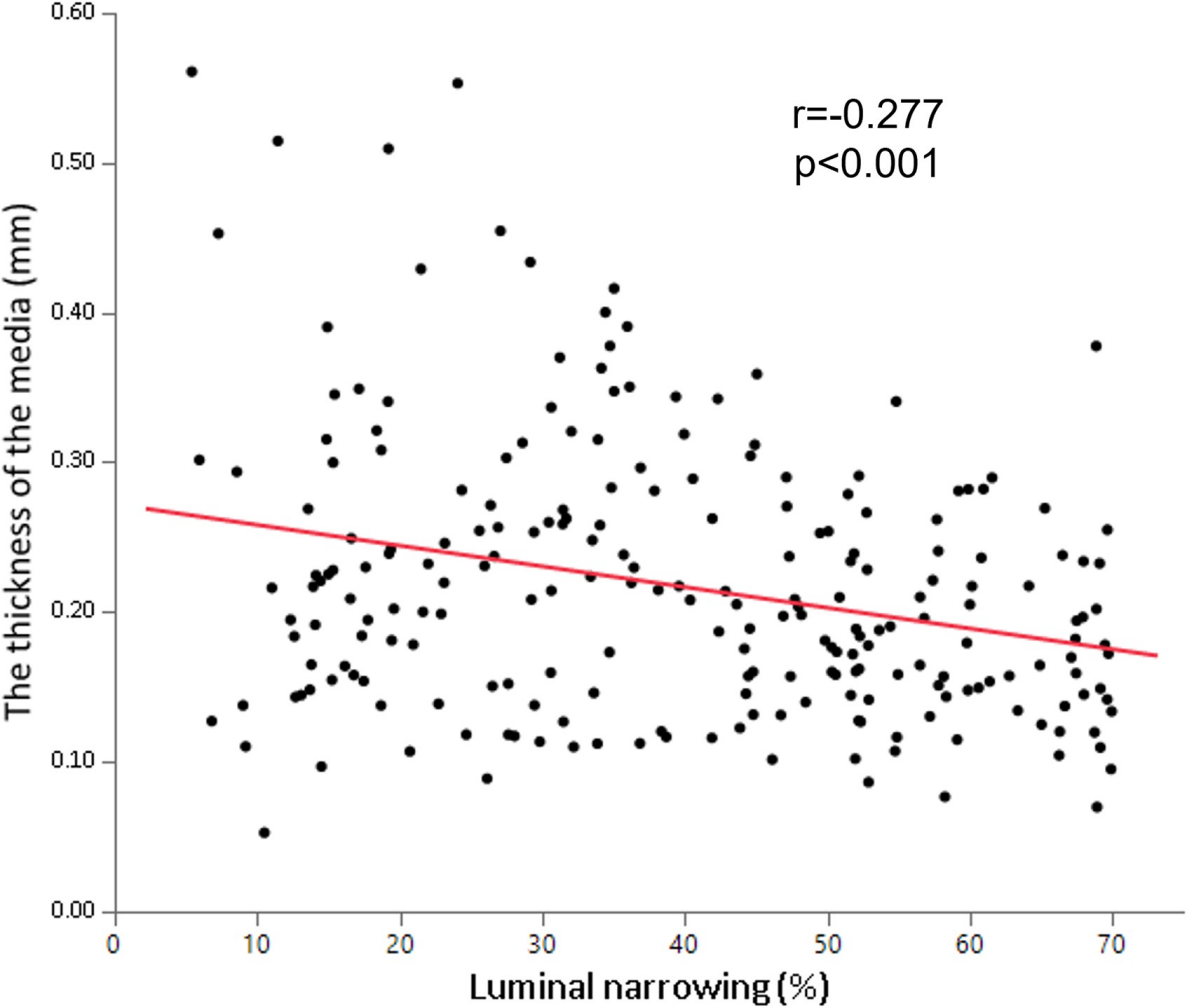
The relationship between the medial thicknesses and the luminal narrowing. The thickness of the media inversely correlated with the luminal narrowing (r = -0.277, p<0.001).

**Fig 5:**
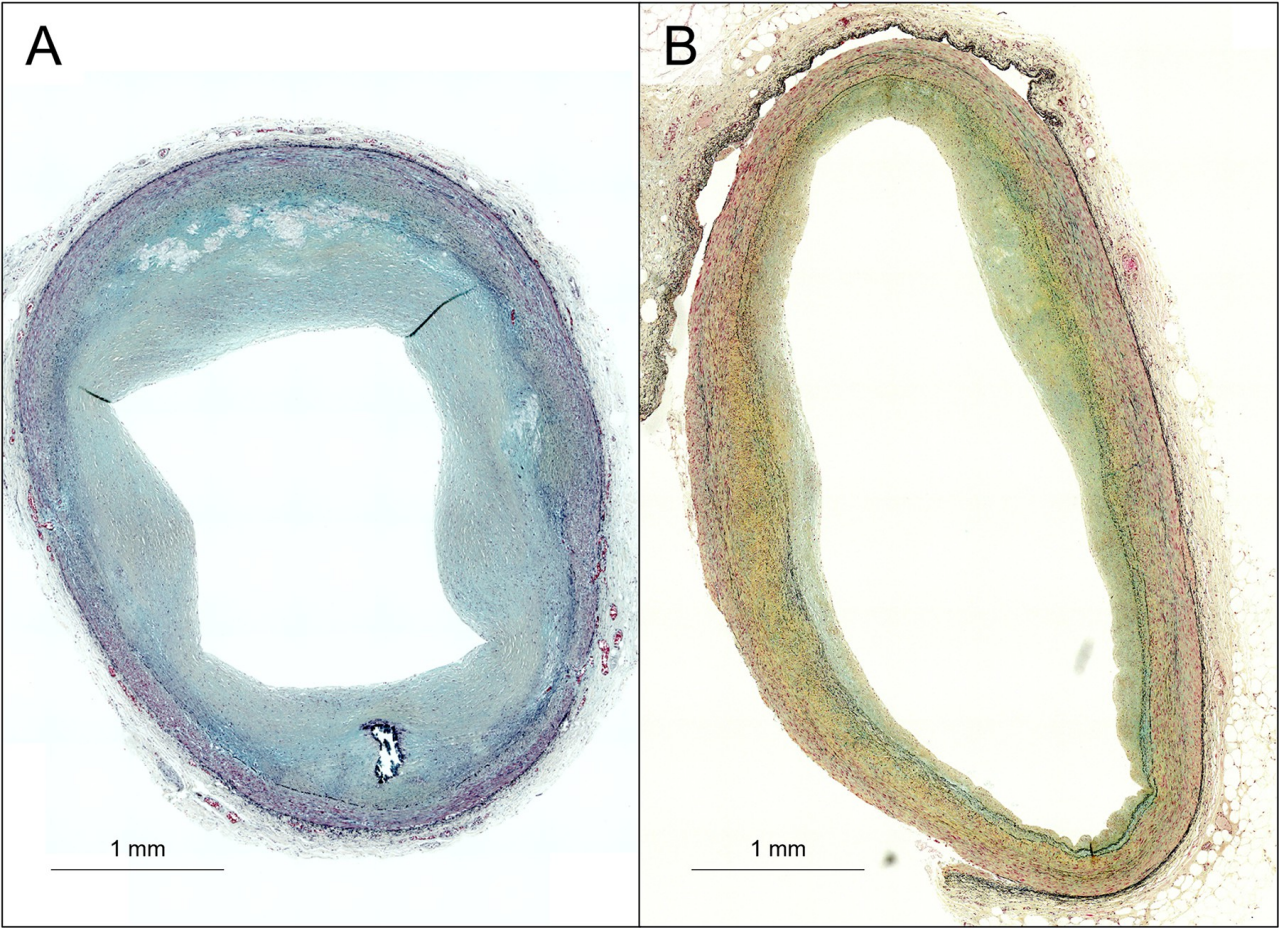
Representative images of sections with different degrees of luminal narrowing. **A, B.** Representative images of the sections in the left circumflex artery in a 63-year-old male. The vessel diameters are almost similar between **A** (3.31mm) and **B** (3.39 mm). However, the luminal narrowing and medial thickness are measured as (A) 60.0% and 0.217 mm and (B) 19.0%, and 0.324 mm, respectively.

There was a positive correlation between the medial thickness and the vessel diameter (r = 0.365, p<0.001) (Fig 3). On the other hand, the medial thickness negatively correlated with luminal narrowing (r = -0.277, p<0.001) (Fig 4). The representative histological images of sections with different degrees of luminal narrowing are shown in Fig 5.

## Discussion

The major observations made in this study are as follows: (i) the medial thickness was positively correlated with the vessel diameter, and (ii) the medial thickness was negatively correlated with the luminal narrowing. To the best of our knowledge, this study was the first to (a) perform detailed measurements of the medial thickness for the proximal, mid, and distal segments at each main coronary artery and side branch artery by pathological analysis, and (b) reveal the association between the medial thickness, vessel diameter, and luminal narrowing. Our work contributes to the basic knowledge of coronary anatomy and to the understanding of the media’s changes in the development of atherosclerosis.

The detailed measurement of the coronary media has not been described thus far. We classified all epicardial coronary arteries into 13 segments, including LMT and side-branch arteries, and performed a more detailed pathological analysis of the medial thickness in non-coronary death patients. A previous report performed histological measurements for medial thickness of LAD, LCX, and RCA, but did not show the measurements of medial thickness for each segment of each epicardial artery [11]. They showed that the average thickness of the coronary artery media in disease-free segments was 0.203 mm. Our results are consistent with this previous report (Table 2).

This study showed that the RCA was less tapered compared to LAD and LCX (S1 Fig), which is consistent with the previous imaging studies [12, 13]. Furthermore, vessel diameter was positively correlated with the medial thickness (Fig 3). Therefore, the medial thickness of RCA was not significantly decreased from the proximal to the distal segment (Fig 2C) while significant decreases in the medial thickness were observed in LAD and LCX (Fig 2A and Fig 2B).

The medial thickness was negatively correlated with luminal narrowing (Fig 4), which suggests that medial thinning is associated with atherosclerotic development. The previous study showed that the average thickness of the coronary artery media was thinner in atherosclerotic segments (mean 88.4 μm) compared to non-atherosclerotic segments (mean 202.9 μm) in the same vessel [11]. Another study indicated that the severity of intimal lesions was related to a significant increase in medial inflammation and vascularization, with a significant decrease in medial thickness [14]. Further studies are needed to understand the mechanisms of medial thinning during atherosclerotic development.

Our study has a substantial impact on clinical practices and future research. Firstly, the understanding of basic anatomy of coronary media may be useful for improving the success rate and predicting risk during the PCI. Due to technological advances in PCI, various devices have increased the procedural success rate for complex lesions. The antegrade dissection and reentry (ADR) device is one of them. A recent study suggested that the successful re-entry point of ADR was significantly likely to be at a site with no plaque or calcification [15]. When the puncture point has less plaque at the distal true lumen in the pre-procedural CCTA, it is useful to predict the medial thickness beforehand and to recognize it during the CTO-PCI procedure, possibly facilitating dissection re-entry. Furthermore, one of the potential risks of using rotational atherectomy is coronary perforation. Although the incidence of coronary perforation during rotational atherectomy or orbital atherectomy is estimated to be 1.5–1.7% in contemporary PCI [16, 17], when it occurs, this can lead to fatal complications, including cardiac tamponade. Because it is often difficult to prevent the plaque ablation catheter from contacting healthy portions at every ablation site, particularly in a tortuous vessel with eccentric plaque, the knowledge of medial thickness at the target vessel should be useful for securing the safety of PCI. A previous study showed that the side-branch rotablation was associated with a higher incidence of coronary perforation during the PCI, compared to main-branch rotablation (6.3% vs. 0.8%, respectively, P = 0.041) [18]. This may be partially explained by our result that a side branch has a thinner media compared to a main branch (Fig 1). Therefore, we should be more cautious when treating a side-branch calcified lesion by rotational atherectomy. Secondly, coronary medial thickness can provide clinically important information for the prevention of restenosis after PCI. Histological studies suggest that medial damage or disruption was a high-risk of restenosis after stent implantation [9, 10]. As patients with a thinner coronary media may tend to have medial disruption caused by dilatation of balloon or stent during PCI, they need more intensive management of other risk factors after PCI. Thirdly, recent studies using animal models have shown that smooth muscle cells migrate from media to intima and change the phenotypes into macrophages or fibroblasts as atherosclerosis progresses [19, 20]. The significant association between luminal narrowing and medial thinning in this study could be important evidence in the future study investigating the mechanism of medial thinning in human coronary arteries.

### Study limitations

The sample size of this study, which was limited to autopsy cases from patients who died of non-coronary deaths, was small. Although patients who died from non-coronary deaths were randomly selected from our registry spanning over 20 years, there could potentially be a selection bias. However, we believe that the sectioning at every 5 mm starting from the proximal segment extending to the distal segment in each vessel made the sampling fairly representative. We also believe that the number of sections evaluated is large enough to analyze the medial thickness in each coronary artery in subjects dying with non-coronary artery disease. Detailed clinical information (including past medical history) was not available in some cases. As this study included only subjects with luminal narrowing of less than 70%, the results of the current study should be confirmed in subjects with more advanced atherosclerosis. However, this study for the first time provides detailed measurements of medial thickness at various segments of coronary arteries from an established autopsy registry, and grants useful insight into the relationship between medial thinning and atherosclerosis.

## Conclusions

Knowledge of coronary medial thickness is important for understanding the basic coronary anatomy and the association of coronary media with atherosclerotic development. The coronary arteries demonstrate variation in medial thickness (varying depending upon the epicardial coronary artery itself, including its segments and branches). Understanding these variations in medial thickness can be useful for both interventionalists and interventional product development teams.

## Supporting information

S1 Table Patient characteristics. (DOCX)

S2 Table The comparison of luminal narrowing and plaque type between main and side branches. (DOCX)

S3 Table The comparison of luminal narrowing and plaque type between proximal, mid, and distal left anterior descending arteries. (DOCX)

S4 Table The comparison of luminal narrowing and plaque type between proximal, mid, and distal left circumflex arteries. (DOCX)

S5 Table The comparison of luminal narrowing and plaque type between proximal, mid, and distal right coronary arteries. (DOCX)

S1 Fig The comparison of the tapered ratio of the main branches. (TIF)
