# Supplementary material for: The histological analysis of the coronary medial thickness: Implications for percutaneous coronary intervention
Source: PLoS One. 2023 Mar 31;18(3):e0283840. doi: 10.1371/journal.pone.0283840 (PMC10065270; doi:10.1371/journal.pone.0283840)
Supplement: S1 Table — (DOCX) [file pone.0283840.s001.docx]

**S1 Table. Patient characteristics**

| Patient, n | n=10 |
| --- | --- |
| Age, years | 60.4 ± 12.3 |
| Male sex, n (%) | 5 (50) |
| Body height (cm) | 170.7±8.9 |
| Body weight (kg) | 84.1±16.9 |
| Body mass index (kg/m^2^) | 28.9±6.0 |
| Heart weight (g) | 512±147 |

Continuous variables are presented as mean ± standard deviation if normally distributed and median (interquartile range) if not normally distributed.
