# Supplementary material for: The histological analysis of the coronary medial thickness: Implications for percutaneous coronary intervention
Source: PLoS One. 2023 Mar 31;18(3):e0283840. doi: 10.1371/journal.pone.0283840 (PMC10065270; doi:10.1371/journal.pone.0283840)
Supplement: S2 Table — (DOCX) [file pone.0283840.s002.docx]

**S3 Table. The comparison of luminal narrowing and plaque type between main and side branches**

| Sections (n=230) | Main (n=190) | Side (n=40) | P value |
| --- | --- | --- | --- |
| Luminal narrowing (%) | 41.9 (26.3-54.8) | 29.6 (19.0-52.9) | 0.083 |
| Plaque type | | | 0.20 |
| AIT, n (%) | 87 (46) | 22 (55) |  |
| PIT, n (%) | 49 (26) | 13 (33) |  |
| Fibroatheroma, n (%) | 5 (3) | 0 |  |
| Fibrocalcific, n (%) | 49 (26) | 5 (13) |  |

Continuous variables are presented as mean ± standard deviation if normally distributed and median (interquartile range) if not normally distributed. AIT, adaptive intimal thickening; PIT, pathological intimal thickening.
