# Supplementary material for: The histological analysis of the coronary medial thickness: Implications for percutaneous coronary intervention
Source: PLoS One. 2023 Mar 31;18(3):e0283840. doi: 10.1371/journal.pone.0283840 (PMC10065270; doi:10.1371/journal.pone.0283840)
Supplement: S3 Table — (DOCX) [file pone.0283840.s003.docx]

**S4 Table. The comparison of luminal narrowing and plaque type between proximal, mid, and distal left anterior descending arteries**

| Sections (n=55) | PLAD (n=25) | MLD (n=15) | DLAD (n=15) | P value |
| --- | --- | --- | --- | --- |
| Luminal narrowing (%) | 36.5±21.5 | 51.3±10.7 | 40.5±18.4 | 0.054 |
| Plaque type | | | | 0.176 |
| AIT, n (%) | 13 (52) | 4 (27) | 8 (53) |  |
| PIT, n (%) | 2 (8) | 5 (33) | 4 (27) |  |
| Fibroatheroma, n (%) | 0 | 0 | 0 |  |
| Fibrocalcific, n (%) | 10 (40) | 6 (40) | 3 (20) |  |

Continuous variables are presented as mean ± standard deviation if normally distributed and median (interquartile range) if not normally distributed. PLAD, proximal left anterior descending artery; MLAD, mid left anterior descending artery; DLAD, distal left anterior descending artery; AIT, adaptive intimal thickening; PIT, pathological intimal thickening.
