# Supplementary material for: The histological analysis of the coronary medial thickness: Implications for percutaneous coronary intervention
Source: PLoS One. 2023 Mar 31;18(3):e0283840. doi: 10.1371/journal.pone.0283840 (PMC10065270; doi:10.1371/journal.pone.0283840)
Supplement: S4 Table — (DOCX) [file pone.0283840.s004.docx]

**S5 Table. The comparison of luminal narrowing and plaque type between proximal, mid, and distal left circumflex arteries**

| Sections (n=52) | PLC (n=25) | MLC (n=15) | DLC (n=12) | P value |
| --- | --- | --- | --- | --- |
| Luminal narrowing (%) | 28.6 (15.7-47.4) | 43.7±13.0 | 42.0±20.7 | 0.060 |
| Plaque type | | | | 0.111 |
| AIT, n (%) | 14 (56) | 6 (40) | 5 (42) |  |
| PIT, n (%) | 2 (8) | 6 (40) | 4 (33) |  |
| Fibroatheroma, n (%) | 0 | 0 | 1 (8) |  |
| Fibrocalcific, n (%) | 9 (36) | 3 (20) | 2 (17) |  |

Continuous variables are presented as mean ± standard deviation if normally distributed and median (interquartile range) if not normally distributed. PLC, proximal left circumflex artery; MLC, mid left circumflex artery; DLC, distal left circumflex artery; AIT, adaptive intimal thickening; PIT, pathological intimal thickening.
