# Supplementary material for: The histological analysis of the coronary medial thickness: Implications for percutaneous coronary intervention
Source: PLoS One. 2023 Mar 31;18(3):e0283840. doi: 10.1371/journal.pone.0283840 (PMC10065270; doi:10.1371/journal.pone.0283840)
Supplement: S5 Table — (DOCX) [file pone.0283840.s005.docx]

**S6 Table. The comparison of luminal narrowing and plaque type between proximal, mid, and distal right coronary arteries**

| Sections (n=64) | PRC (n=28) | MRC (n=18) | DRC (n=18) | P value |
| --- | --- | --- | --- | --- |
| Luminal narrowing (%) | 40.8±19.2 | 52.3±13.8 | 43.4 (33.6-58.7) | 0.133 |
| Plaque type | | | | 0.20 |
| AIT, n (%) | 13 (46) | 5 (28) | 7 (39) |  |
| PIT, n (%) | 8 (29) | 5 (28) | 8 (44) |  |
| Fibroatheroma, n (%) | 0 | 2 (11) | 2 (11) |  |
| Fibrocalcific, n (%) | 7 (25) | 6 (33) | 1 (6) |  |

Continuous variables are presented as mean ± standard deviation if normally distributed and median (interquartile range) if not normally distributed. PRC, proximal right coronary artery; MRC, mid right coronary artery; DRC, distal right coronary artery; AIT, adaptive intimal thickening; PIT, pathological intimal thickening.
