# Supplementary figures and images for: The histological analysis of the coronary medial thickness: Implications for percutaneous coronary intervention
Source: PLoS One. 2023 Mar 31;18(3):e0283840. doi: 10.1371/journal.pone.0283840 (PMC10065270; doi:10.1371/journal.pone.0283840)

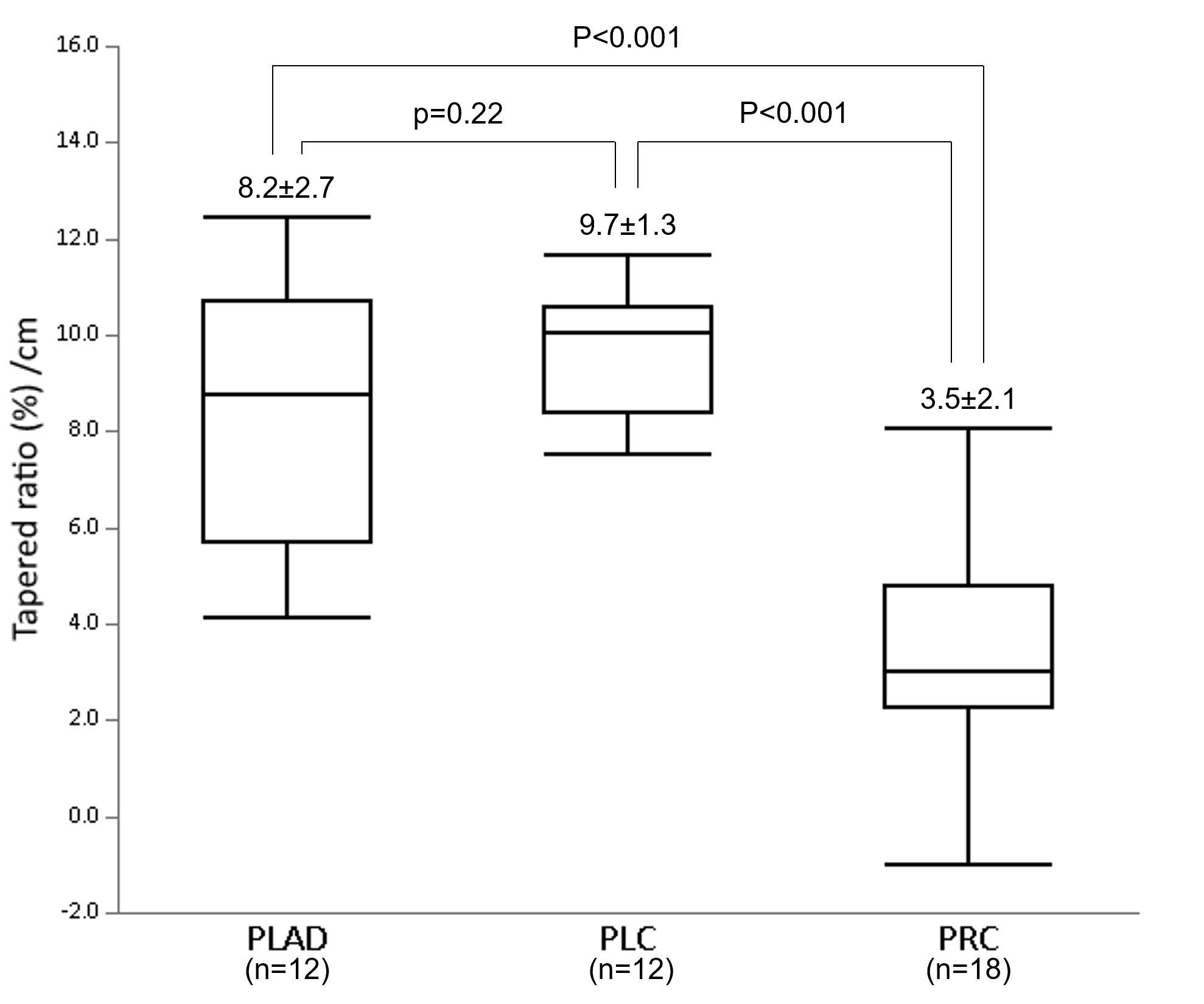

Supplement: S1 Fig — (TIF) [file pone.0283840.s006.tif]
